# Supplementary material for: Causal Relationship between Aging and Anorexia Nervosa: A White-Matter-Microstructure-Mediated Mendelian Randomization Analysis
Source: Biomedicines. 2024 Aug 16;12(8):1874. doi: 10.3390/biomedicines12081874 (PMC11351342; doi:10.3390/biomedicines12081874)
Supplement: Supplementary file 1 [file biomedicines-12-01874-s001.zip › Supplementary Figure/Figure S4/Singe SNP plot of MR analysis for TL to ubm-b-1720.pdf]

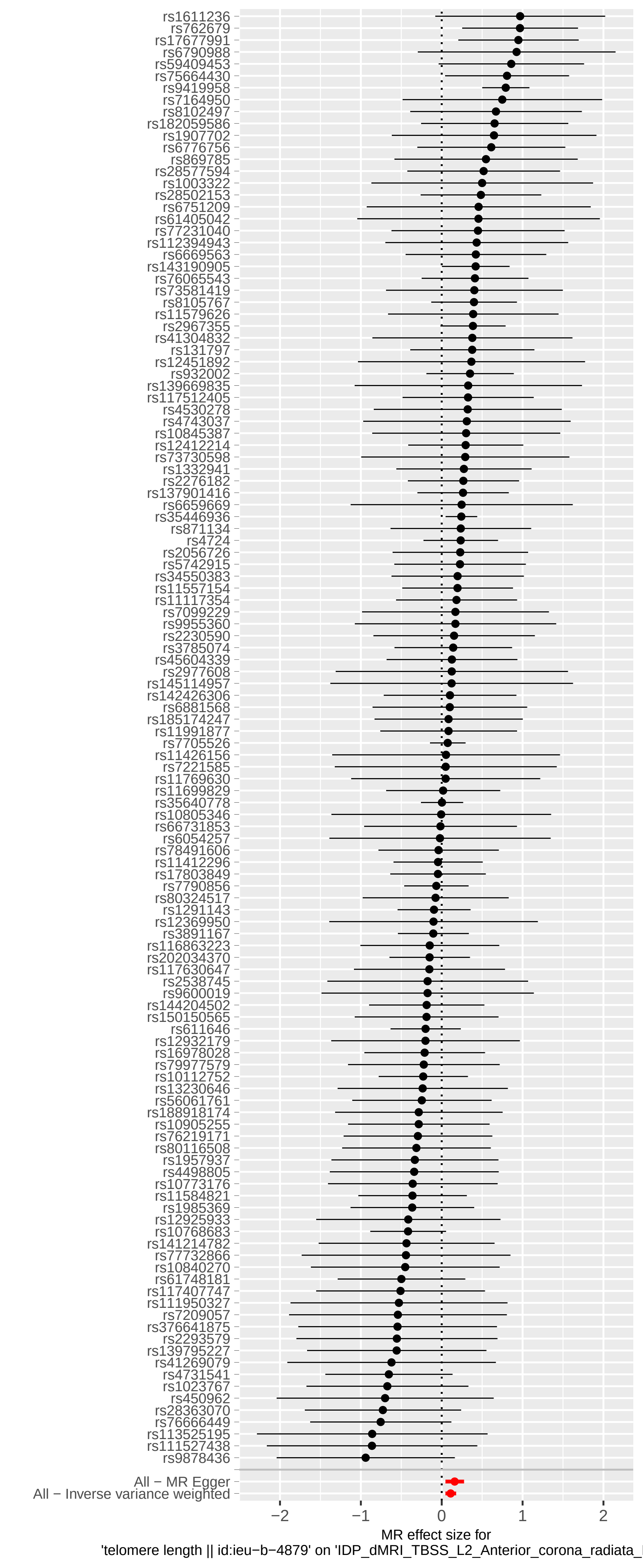
rs1611236
rs762679
rs17677991
rs6790988
rs59409453
rs75664430
rs9419958
rs7164950
rs8102497
rs182059586
rs1907702
rs6776756
rs869785
rs28577594
rs1003322
rs28502153
rs6751209
rs61405042
rs77231040
rs112394943
rs6669563
rs143190905
rs76065543
rs73581419
rs8105767
rs11579626
rs2967355
rs41304832
rs131797
rs12451892
rs932002
rs139669835
rs117512405
rs4530278
rs4743037
rs10845387
rs12412214
rs73730598
rs1332941
rs2276182
rs137901416
rs6659669
rs35446936
rs871134
rs4724
rs2056726
rs5742915
rs34550383
rs11557154
rs11117354
rs7099229
rs9955360
rs2230590
rs3785074
rs45604339
rs2977608
rs145114957
rs142426306
rs6881568
rs185174247
rs11991877
rs7705526
rs11426156
rs7221585
rs11769630
rs11699829
rs35640778
rs10805346
rs66731853
rs6054257
rs78491606
rs11412296
rs17803849
rs7790856
rs80324517
rs1291143
rs12369950
rs3891167
rs116863223
rs202034370
rs117630647
rs2538745
rs9600019
rs144204502
rs150150565
rs611646
rs12932179
rs16978028
rs79977579
rs10112752
rs13230646
rs56061761
rs188918174
rs10905255
rs76219171
rs80116508
rs1957937
rs4498805
rs10773176
rs11584821
rs1985369
rs12925933
rs10768683
rs141214782
rs77732866
rs10840270
rs61748181
rs117407747
rs111950327
rs7209057
rs376641875
rs2293579
rs139795227
rs41269079
rs4731541
rs1023767
rs450962
rs28363070
rs76666449
rs113525195
rs111527438
rs9878436
All − MR Egger
All − Inverse variance weighted
−2
−1
0
1
2
MR effect size for
'telomere length || id:ieu−b−4879' on 'IDP_dMRI_TBSS_L2_Anterior_corona_radiata_
